# Potential of Image2Image translation in reducing AI bias attributed to differences in CT reconstruction methods: proof-of-concept study on a paired dataset

**DOI:** 10.3389/fradi.2026.1875867

**Published:** 2026-07-03

**Authors:** Feifei Li, Liliana Lourenco Caldeira, Astha Jaiswal, Nils Große Hokamp, Oya Beyan, Ekaterina Kutafina

**Affiliations:** 1 Institute for Biomedical Informatics, University of Cologne, Faculty of Medicine and University Hospital Cologne, Cologne, Germany; 2 Institute for Diagnostic and Interventional Radiology, University of Cologne, Faculty of Medicine and University Hospital Cologne, Cologne, Germany; 3 FAIR Data and Distributed Analytics Department, Fraunhofer Institute for Applied Information Technology, FIT, Aachen, Germany

**Keywords:** bias, CT reconstruction, generative AI, Image2Image translation, machine learning

## Abstract

**Background:**

The choice of image reconstruction approach, such as Iterative Model Reconstruction (IMR) and iDose${}^{4}$, is critical in computed tomography (CT) as distribution shifts between them can introduce systematic bias in clinical AI models, posing a barrier to efficient, scalable AI deployment in radiology. While Image-to-Image (I2I) translation offers a path toward harmonization, efficient and translation-feasible AI in radiology demands that such approaches be validated not only on image quality metrics but also on downstream clinical task performance.

**Methods:**

We compared three harmonization strategies: CycleGAN, Denoising Diffusion Models, and a conventional Gaussian filter, using a paired CT dataset. Performance was evaluated using task-agnostic metrics, including Structural Similarity Index Measure (SSIM), Peak Signal-to-Noise Ratio (PSNR), Frechet Inception Distance (FID), and Sliced Wasserstein Distance (SWD). Crucially, we assessed clinical utility by measuring the downstream impact on multi-organ segmentation using Mean Relative Volume Error (RVE), focusing on consistency across reconstructed modes.

**Results:**

Our findings reveal a significant discrepancy between visual fidelity and clinical utility. While Diffusion and CycleGAN models achieve competitive feature-level similarity, they introduce catastrophic systematic biases in downstream segmentation, particularly in adipose tissues (Body Composition Analysis, BCA), where volume errors can reach −100%, indicating a failure to preserve critical anatomical fat structures. In contrast, the computationally efficient Gaussian filter served as the most robust baseline, maintaining minimal RVE and the highest consistency between IMR and iDose${}^{4}$ modes.

**Conclusions:**

The results suggest that in the context of efficient AI for radiology imaging, where efficiency encompasses not only computational cost but also reliability of clinical outputs, task-agnostic image quality metrics are insufficient proxies for downstream clinical performance. High-parameter generative models may introduce structural losses that remain invisible to such metrics yet critically affect clinical utility. The underperformance of generative approaches observed here is likely attributable to 3D slice inconsistencies during volumetric reconstruction and suboptimal model adaptation, rather than to an inherent limitation of I2I methods, which retain significant potential for mitigating reconstruction-related bias in radiology AI. Future work developing harmonization pipelines must therefore prioritize task-specific, clinically grounded validation as the primary benchmark—and carefully consider whether computationally simpler approaches, such as conventional filtering, may already provide comparably stable harmonization for specific use cases, before resorting to high-parameter generative solutions.

## 1 Introduction

The current development of AI solutions in radiology requires awareness of different types of biases posing risks to algorithm performance and generalizability. The issue of bias in radiology has been extensively studied across various domains of data handling, model development, and performance evaluation. The work (1) emphasizes the need for robust data collection and exploratory analysis to minimize biases, including selection and measurement errors. The following study (2) investigates model development strategies, including data augmentation and adversarial learning, to mitigate overfitting and ensure model fairness. Meanwhile, Faghani et al. (3) focus on performance evaluation, advocating for external validation and the use of tailored metrics to uncover hidden biases. In the editorial paper, Kahn (4) highlights the significance of transparency tools to promote accountability and reproducibility. These studies offer a comprehensive roadmap for bias mitigation and underscore persistent challenges in practical implementation and data accessibility.

CT is a well-established and widely used tool in medical diagnostics, providing non-invasive, high-resolution images of the body’s internal anatomical structures. CT systems utilize a rotating x-ray source and detector array to acquire a series of projections, which are then reconstructed into cross-sectional images using mathematical algorithms. The choice of a specific reconstruction method depends primarily on the ability to reduce radiation dose and deliver acceptable image quality (5). The work of Blaziset al. (6) demonstrated the impact of reconstruction methods on the performance of a deep-learning-based pulmonary nodule CAD system. Such performance degradation is consistent with the broader phenomenon of distribution shift, in which variations in reconstruction parameters alter the data’s statistical properties relative to the model’s training distribution.

**Figure 1:**
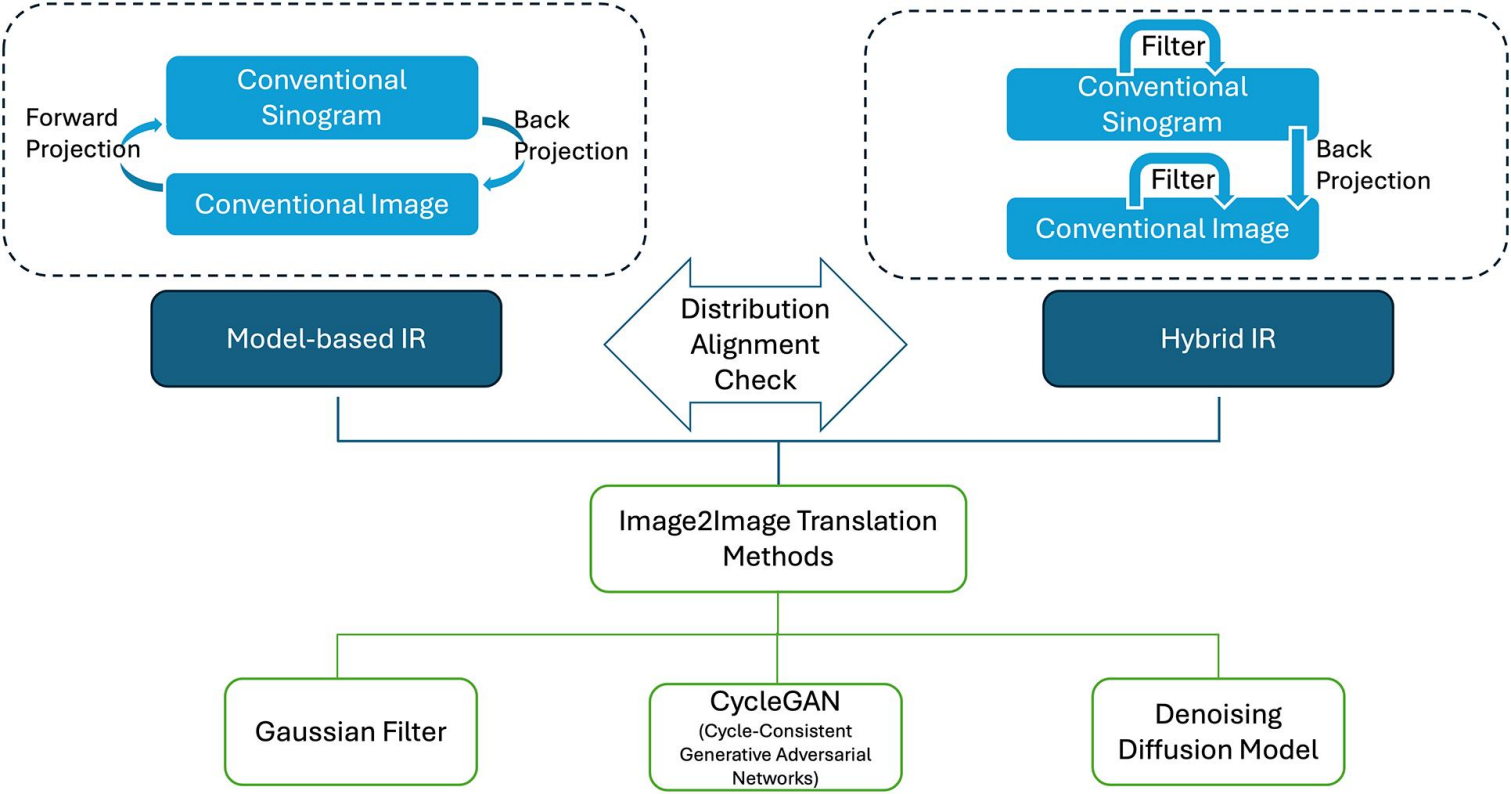
Two different reconstruction methods and Image2Image translation methods: Model-based iterative reconstruction (IR) and Hybrid IR, after distribution alignment examination, processed by the three I2I methods: Gaussian Filter, CycleGAN, and Denoising Diffusion Model.

While the issue of bias in radiology is well-documented, the computational and financial overhead required to adapt models to every specific CT reconstruction kernel remains a significant barrier to efficient deployment. Retraining high-capacity models for each clinical site is often infeasible due to limited labeled data and hardware constraints. Addressing reconstruction-related bias through I2I harmonization is therefore not only a question of diagnostic accuracy, but of deployment efficiency. For a harmonization strategy to be considered efficient in a clinical context, it must satisfy two conditions: it must reduce distribution shift sufficiently to improve downstream task performance, and it must do so with a computational overhead that is compatible with routine clinical workflows. These conditions are not guaranteed by visual similarity alone—a method that produces perceptually convincing outputs may nonetheless fail to preserve the densitometric properties on which downstream clinical algorithms depend. This work addresses the bias resulting from distribution differences linked to the choice of reconstruction methods in CT imaging, as illustrated in Figure 1, and evaluates I2I harmonization strategies explicitly against both conditions.

The rapid development of generative deep learning methods over the past years has enabled the creation of synthetic datasets to address bias in medical imaging (7, 8). While using the generative methods to create the complete synthetic data cohorts might pose challenges to privacy preservation (9) and require a lot of computational resources, the development of “image-to-image translation” (I2I) is becoming a very promising alternative in the cases where we deal with the bias due to the different data modalities (e.g., MRI vs. CT) or due to the differences in reconstruction methods. I2I can, in principle, refer to a wide range of methods of image alteration, such as traditional filters. The first I2I approach based on generative machine learning algorithms was proposed by Isola et al. in 2016 (10), allowing subsequent approaches to focus on image transformation rather than full image synthesis. In medical imaging, I2I solutions have been successfully applied to mitigate distribution-shift bias. A recent paper by Dohmenet al. (11) provides a detailed discussion of I2I approaches to MRI, with a focus on metrics to assess performance and their contextual meaning. The metrics, important for the context of our work, are divided into “direct” ones, assessing the success of the image generation task, and “indirect” ones, focusing on the “core task” algorithm’s (e.g., segmentation) performance on the used dataset.

While many studies address distributional shifts between imaging modalities, such as CT and MRI, the specific bias introduced by differences in reconstruction methods within the same modality has received considerably less attention. In recent years, the only work addressing the bias linked to the differences in reconstruction methods in CT imaging is reported by Krishnan et al. (12). The authors apply a generative I2I method (CycleGAN) to harmonize the data from two different vendors and two reconstruction methods. The results were assessed by comparing the emphysema scores in different data configurations.

Our work analyses the direct, task-agnostic performance of I2I approaches for reconstructing-related bias reduction. We considered two practical use cases in which I2I methods could be applied across differently reconstructed CT datasets.

### 1.1 Use case 1

**Figure 2:**
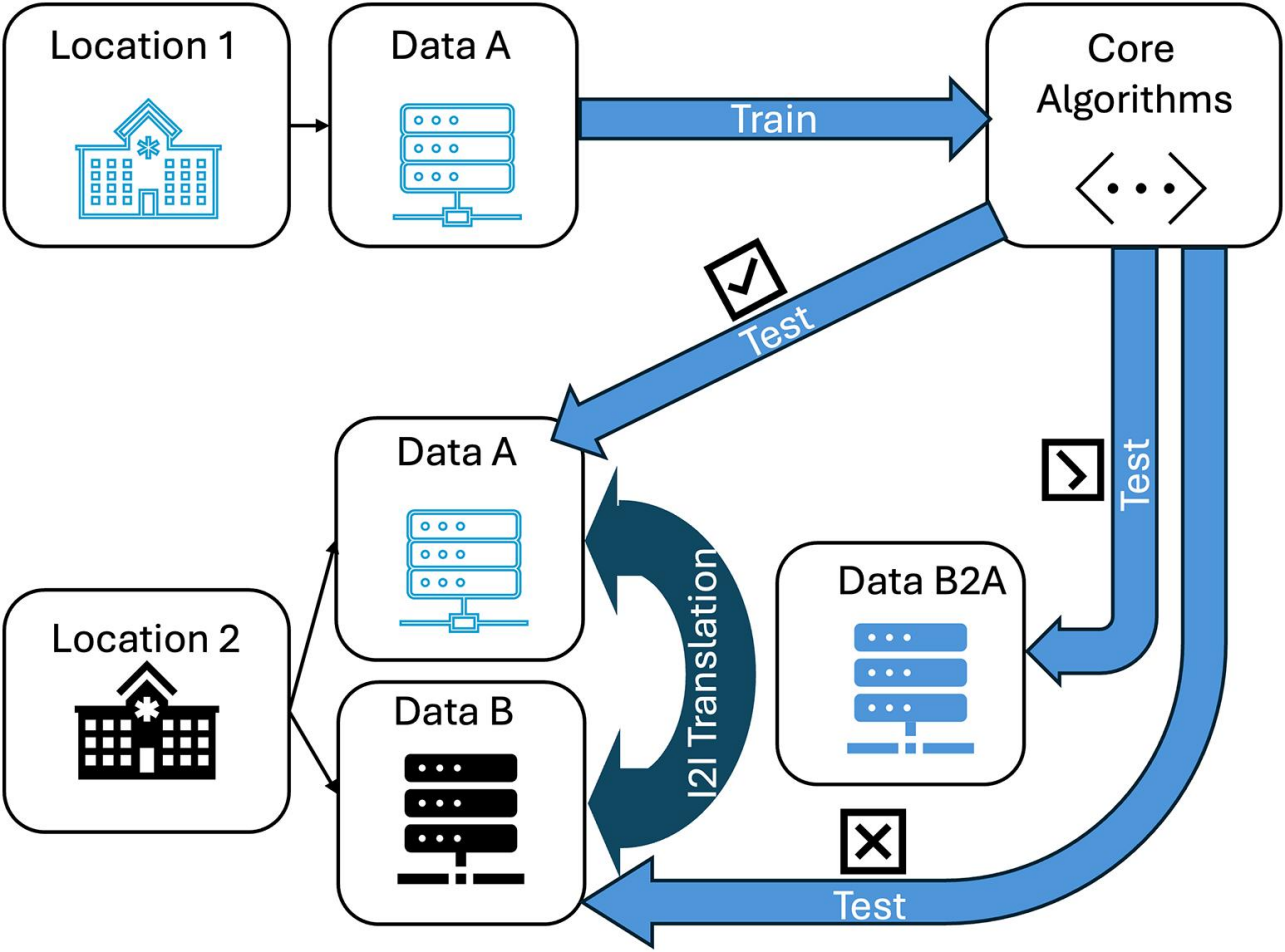
Use Case 1: the applications of I2I translation on different reconstructed methods.

In this Use Case (see Figure 2), the first research group (Location 1) reconstructs the data with method A (Data A) and trains an algorithm on Data A to perform a medically relevant task (Core Algorithms). The second group has access to both Data A (the name refers only to the reconstruction type, not to the dataset’s exact content) and Data B. The core algorithms, trained on Data A, are expected to work well on the first part (A), but may fail on differently distributed Data B. However, local access to both data types allows training a generative algorithm (such as CycleGAN) to translate Data B to Data B2A with a distribution close to Data A, ensuring successful execution of the core task.

### 1.2 Use case 2

**Figure 3:**
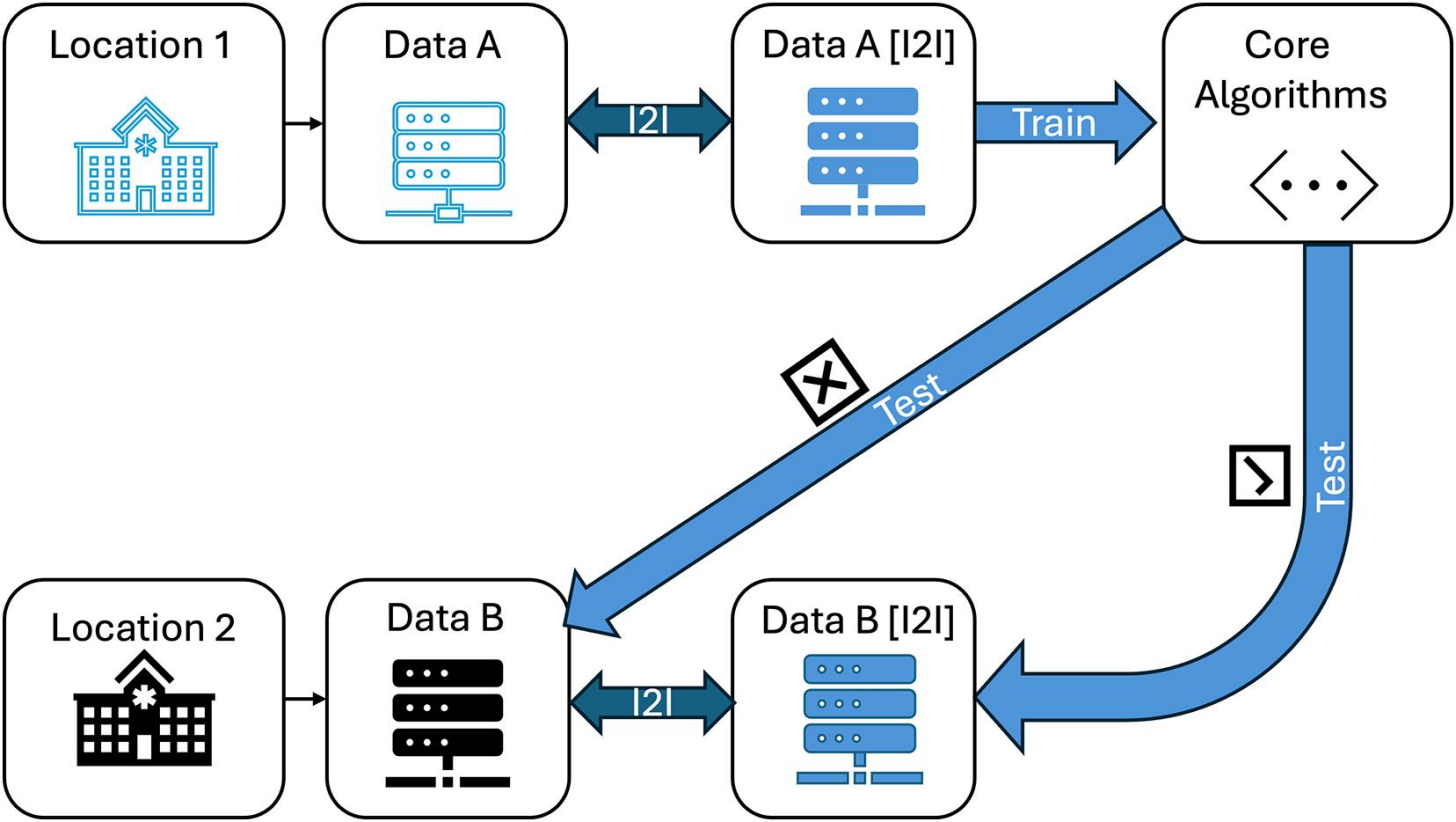
Use Case 2: the applications of I2I translation on different reconstructed methods.

In this Use Case (see Figure 3), two research groups have differently reconstructed data sets (each location has access to only one data type). However, because two different reconstruction methods are applied to the same core resource of x-ray projections, applying denoising methods to both datasets may bring the distributions closer together. Hence, Data A is denoised using a specific I2I algorithm (either based on machine learning or traditional signal processing methods) that does not require access to Data B. The same I2I algorithm is applied independently to Data B. After this preparation, the core task developed on Data A[I2I] can be tested on Data B[I2I].

The choice of methods and metrics was informed by the literature cited above and by the review paper by Hoyez et al. (13). The CycleGAN model, introduced by Zhu et al. (14), remains one of the most successful and widely used approaches. The Denoising Diffusion model (15) is a deep learning algorithm that progressively refines images through a learned denoising process, here applied to reduce reconstruction-induced intensity variations. One important difference in the context of sensitive data is that Diffusion Models can be trained only on the target data (Data B, see Figure 3), whereas the CycleGAN approach requires access to both datasets during training. Finally, Gaussian Filters are applied to achieve transparent, computationally efficient denoising algorithms in signal processing.

This paper contributes to the field by comparing representative I2I harmonization strategies—spanning generative deep learning and classical signal processing approaches—for reducing CT data distribution discrepancies arising from different reconstruction methods. We leverage a paired dataset of CT scans reconstructed with two methods (Philips IMR and iDose${}^{4}$ Ya), collected from 5 patients, enabling pairwise image comparisons in the pixel- and feature-domains. In addition, we assess anatomical structure segmentation using Body Composition Analysis (BCA) and TotalSegmentator (16), providing a downstream clinical validity evaluation alongside task-agnostic distribution metrics.

## 2 Methods

**Figure 4:**
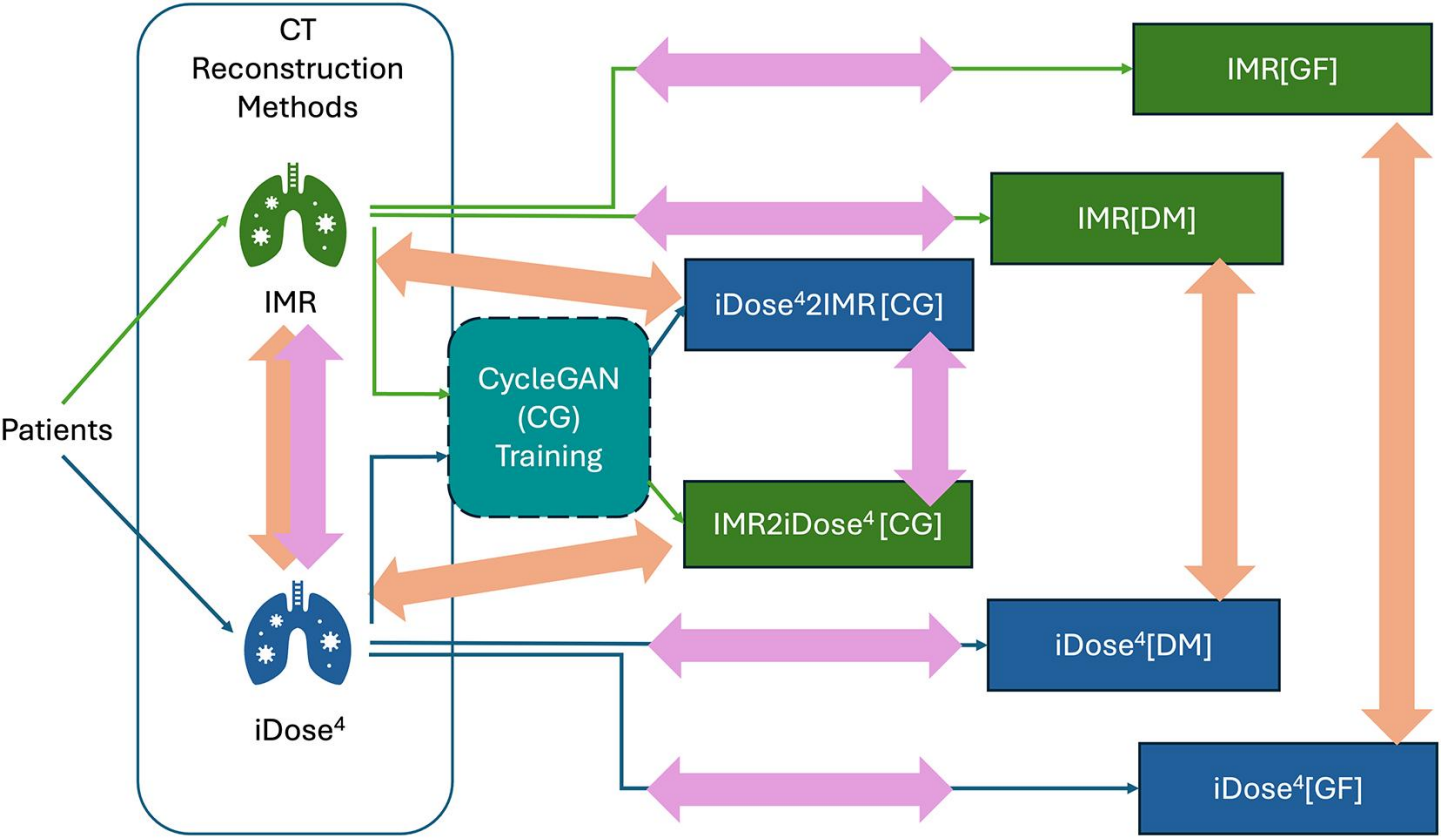
Distribution similarity check flow. The patients’ CT scans are reconstructed with two methods: IMR (green) and iDose${}^{4}$ with Ya Kernel (blue). Both datasets are denoised with the Gaussian Filter (GF) and Denoising Diffusion model (DM). Both data are used to train CycleGAN for cross-translating the data: e.g., IMR is translated to iDose${}^{4}$, denoted by IMR2iDose${}^{4}$ [CG] and vice versa. The broad arrows indicate the performed comparisons between the datasets. Orange denotes comparisons relevant to the potential performance of the core model in the Use Cases, while pink denotes comparisons aimed at understanding the scale of data alterations associated with each method.

In our study, we use retrospective paired CT scans to identify pairwise differences between datasets as a proxy, algorithm-agnostic measure of potential core-algorithm bias. Further, using I2I methods, we harmonize the data from the two reconstruction methods and assess improvements in distributional differences. The workflow is presented in Figure 4. After the distribution comparisons, we apply a segmentation downstream task to analyze the differences of BCA and organ volumes.

### 2.1 Data and reconstruction methods

This study utilizes a dataset of five paired patient CT scans, recorded with Philips IQon Spectral CT, at the Radiology Department of the University Hospital of Cologne during routine diagnostic procedures and reconstructed with two different methods: The Iterative Model Reconstruction (IMR) and the hybrid algorithm iDose${}^{4}$ with Ya Kernel (17). Data for this retrospective analysis were extracted in NIfTI format and limited to pseudonymized torso images acquired during routine clinical practice.

We follow the choice presented in the publications such as (12, 18), and work with 2D slices. After slicing the 3D scans into 2D images, the number of slices for five paired patients is as follows: patient 1: 254, patient 2: 281, patient 3: 302, patient 4: 341, and patient 5: 313. There are 1491 slices in total, each with a resolution of 512×512.

The choice of a 2D Image-to-Image (I2I) translation pipeline instead of a volumetric 3D architecture is informed by both computational and structural considerations, aligning with our previous findings (19). Training deep generative models (such as CycleGAN or Diffusion models) directly on dense 3D CT volumes introduces prohibitive computational overhead and memory constraints. To mitigate this, 3D networks typically rely on small-patch-based training. However, patch-based 3D generation frequently introduces stitching artifacts and severe boundary discontinuities across neighboring patches, which can unpredictably distort critical anatomical structures and degrade downstream multi-organ segmentation performance. To address these challenges within an ’efficient AI’ framework, our study deliberately set out to evaluate a lighter, more computationally accessible 2D slice-by-slice pipeline as an alternative, capitalizing on its superior algorithmic stability and high intra-slice texture fidelity. The potential trade-offs of this 2D strategy regarding inter-slice continuity along the Z-axis and its subsequent impact on downstream volumetric quantification are further detailed in the Discussion section.

An example of paired image slices from the same patient is shown in the first column of the Figure 6. The visible variations between the images highlight potential sources of computational bias, which we assess in this study.

### 2.2 I2I models and training

CycleGAN, a deep learning-based approach using Generative Adversarial Networks (GANs), was originally developed to learn mappings between different image domains in an unpaired setting (14). Using two networks (a generator and a discriminator), CycleGAN learns to translate images between the domains. For our project, we utilize the publicly available PyTorch implementation https://github.com/junyanz/pytorch-CycleGAN-and-pix2pix, from the paper (20). The CycleGAN algorithm can be used for unaligned data translation by adding an identity loss. The selection of CycleGAN as the primary generative baseline, despite the availability of a perfectly paired dataset, is justified by two factors. First, from a clinical translation perspective, perfectly paired projection data is rarely available across multi-center environments; thus, evaluating an unpaired-capable architecture provides a more realistic and scalable benchmark for real-world deployment. Second, this architecture was selected to maintain direct methodological consistency with our previously established 3D medical image harmonization workflows (19), enabling a controlled comparison between 2D and 3D paradigms under identical cyclic loss constraints. Nevertheless, we acknowledge that the weaker cyclic and adversarial constraints of CycleGAN, compared to the rigid pixel-wise $L_{1}$ loss of paired networks like Pix2Pix, may inherently permit the localized intensity drift observed in low-density tissues.

**Figure 5:**
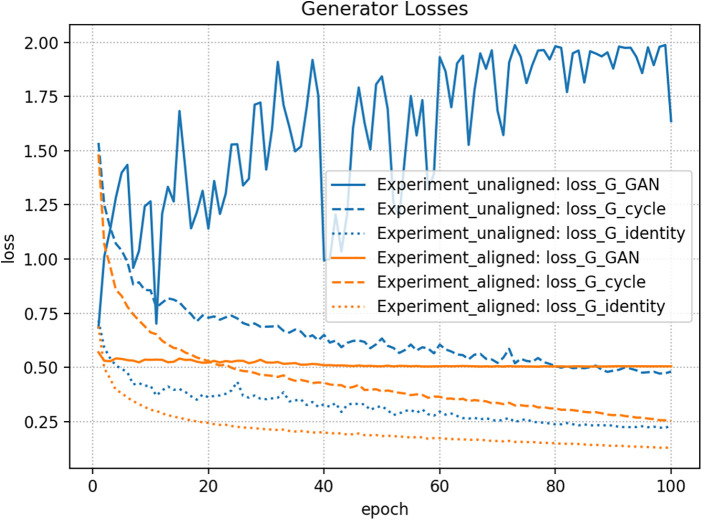
Unaligned and aligned CycleGAN training results. The loss values are computed and averaged across our five patients, using five-fold validation (a training/test ratio of 4:1). Loss-G-GAN denotes the adversarial loss, measuring how well the generator fools the discriminator into believing the generated images are real. Loss-G-cycle denotes the cycle-consistency loss, measuring how well an image can be translated to another domain and back to its original form. Loss-G-identity denotes the identity loss, measuring the extent to which the generator changes images that already belong to the target domain.

We evaluated the impact of data alignment on model performance, as illustrated in Figure 5. Our results demonstrate that the aligned setting—where training pairs consist of corresponding slice indices—yielded significantly accelerated convergence compared to the unaligned setting, in which slices were paired stochastically.

Using aligned data makes it easier to achieve stable performance, which not only reduces training time but also better guides CycleGAN to learn the characteristic intensity and texture differences between the two CT reconstruction methods. Hence, we use the same hyperparameter settings and align the slices from the same patient to train the model using a leave-one-out approach. The overall objective function (Equation 1) for the network training is formulated as a weighted combination of the adversarial loss ($L_{\text{GAN}}$), cycle-consistency loss ($L_{\text{cyc}}$), and identity mapping loss ($L_{\text{id}}$):

$$L_{\text{total}}=\lambda_{\text{GAN}}L_{\text{GAN}}+\lambda_{\text{cycle}}L_{\text{cyc}}+\lambda_{\text{identity}}L_{\text{id}}$$ (1)

To ensure stable convergence, the hyperparameter weights were rigidly set to $\lambda_{\text{GAN}}=1.0$, $\lambda_{\text{cycle}}=10.0$, and $\lambda_{\text{identity}}=5.0$. The loss curves tracking these training dynamics are illustrated in Figure 5.

The Denoising Diffusion Model (15) is a probabilistic framework for progressive denoising that gradually transforms images through a series of learned denoising steps, progressively transforming image distributions toward a target domain in a controlled manner.

For our work, we use a pre-trained Denoising Diffusion Model (21) available in the repository at https://github.com/DeepXuan/Dn-Dp. This model utilises only normal-dose CT images during training, enabling zero-shot denoising of low-dose CT images. Since the pre-trained model was trained on chest CT data comparable to ours, only the batch size was adjusted to 2 to accommodate the available hardware (16 GB GPU, NVIDIA A400).

In the leave-one-out training, we first use the network with the “chest-256×256” set of parameters on our slices, then apply the “chest-256×256-512×512” model.

A Gaussian filter with a 5×5 kernel size and standard deviation σ=0 was included as a computationally efficient, parameter-free baseline, representing classical signal-processing harmonization against which the generative approaches are compared. In the Python OpenCV library, if σ=0, according to the standard algorithmic framework, σ=0.3×((ksize−1)×0.5−1)+0.8, ksize=5 yielded a fixed standard deviation of σ=1.1, precisely adjusting the smoothing scale to the pixel granularity of the slices without manual bias.

### 2.3 Evaluation

In our evaluation process, we consider two types of comparisons. The primary objective of applying I2I translations is that the core models in both Use Cases increase their chances of performing well on the test data. In Use Case 1 Figure 2, Data B2A should have the distribution closer to Data A, compared to Data B. In Use Case 2 Figure 3, Data A[I2I] and B[I2I] are expected to have closer distributions than the original Data A and B. We use four metrics to assess the reduction in distribution shift: Structural Similarity Index Measure (SSIM), Peak Signal-to-Noise Ratio (PSNR), Fréchet Inception Distance (FID), and sliced Wasserstein Distance (SWD). The first two metrics work with pixel-to-pixel similarity, and the other two with feature space similarity. This series of measurements can be summarized as “bias reduction potential” and is denoted by orange arrows in the Figure 4.

In addition, to fully comprehend the alterations done by the I2I methods, we also compare different versions of the same data. Those measurements can be grouped as “alteration tracking” and contribute to better interpretability of the applied I2I approaches (pink arrows in Figure 4).

#### 2.3.1 I2I performance metrics

PSNR (22) and SSIM (23) are calculated to quantify structural preservation. For f(i,j),g(i,j) being the pixel values from the 2D data we compute MSE (Mean Squared Error) (Equation 2)

$$\text{MSE}=\frac{1}{mn}\sum_{0}^{m-1}\sum_{0}^{n-1}\|f(i,j)-g(i,j){\|}^{2}$$ (2)

and define PSNR as (Equation 3):

$$\text{PSNR}=20\log_{10}(\frac{\max_{f}}{\sqrt{\text{MSE}}})$$ (3)

The SSIM index (Equation 4) is calculated window-wise. The measure between two windows x and y of the common size N×N is:

$$\text{SSIM( x, y) }=\frac{\left(2\mu_{x}\mu_{y}+c_{1})(2\sigma_{xy}+c_{2}\right)}{\left(\mu_{x}^{2}+\mu_{y}^{2}+c_{1})(\sigma_{x}^{2}+\sigma_{y}^{2}+c_{2}\right)}$$ (4)

where: $\mu_{x,y}$ are the pixel sample means of x and y respectively; $\sigma_{x^{2},y^{2}}$ are the pixel sample variances of x and y; $\sigma_{y}^{2}$ is the pixel sample variance of y; $\sigma_{xy}$ is the pixel covariance of x and y; $c_{1,2}=(k_{1,2}L{)}^{2}$ are two variables to stabilize the division with weak denominator; L the dynamic range of the pixel-values and the default values are set to $k_{1}=0.01$, $k_{2}=0.03$, N=11.

Fréchet inception distance (FID) was introduced by Heusel et al. (24) and measures the discrepancy between the feature distributions of two image sets, using a pre-trained Inception v3 (25) classifier as the feature-extraction backbone. Here, it is used to quantify the distributional distance between differently reconstructed CT datasets. Lower values indicate greater distributional similarity.

The two features’ distribution can be represented as N(μ,Ξ) and $N(\mu^{\prime },\Xi^{\prime })$. The distance (Equation 5) between these two distributions is calculated as the special case of the Wasserstein distance.

$$d_{F}(N(\mu ,\Xi ),N(\mu^{\prime },\Xi^{\prime }){)}^{2}=\|\mu -\mu^{\prime }{\|}_{2}^{2}+tr(\Xi +\Xi^{\prime }-2(\Xi \Xi^{\prime }{)}^{1/2})$$ (5)

The FID measures how often the same high-level features occur in the two image sets.

Sliced Wasserstein Distance (SWD) (Equation 6) was proposed by Karras et al. (26) as a more computationally efficient distance measure. The idea underlying the SWD is to decompose the challenging estimation of a high-dimensional distribution into a simpler estimation of multiple one-dimensional distributions. $P_{DataA}$, $P_{DataB}$ are the probability distributions of random variables and feature distributions after the VGG16 network.

$$\begin{array}{rl} & SWD_{p}(P_{DataA},P_{DataB})\\ & \;={\left(\int_{S^{n-1}}W_{p}(\pi_{\theta }^{*}P_{DataA},\pi_{\theta }^{*}P_{DataB}{)}^{p}\;d\theta \right)}^{1/p} \end{array}$$ (6)

For a unit vector $\theta \in S^{n-1}$, and the corresponding inner product $\pi_{\theta }(x)=\theta^{T}x$ and marginal distribution $\pi_{\theta }^{*}P_{DataA}=P_{DataA}\circ \pi_{\theta }^{-1}$. The lower SWD value indicates higher similarity.

#### 2.3.2 Automated anatomical segmentation and volumetric evaluation

To rigorously assess the anatomical fidelity and clinical utility of the harmonized CT images, we implemented a dual-stage automated segmentation and body composition pipeline. This framework serves as a “downstream task” validation, ensuring that the generative models preserve the precise densitometric and geometric properties required for medical diagnostics. For the segmentation experiments, we converted all 2D slices into 3D representations for organ and tissue segmentation.

##### 2.3.2.1 Intensity Preprocessing and HU Restoration Pipeline

To accommodate the standard architectures of the 2D generative models (CycleGAN and Denoising Diffusion Models), the input CT data required an intensity mapping pipeline. The original clinical CT volumes in NIfTI format contain true Hounsfield Units ($HU_{\text{orig}}$). During preprocessing, a dynamic range clipping window of [−1,024,+3,000]HU was first applied to eliminate extreme outliers while preserving the entire diagnostic spectrum from air to dense bone. These clipped values were then linearly normalized to a standard 8-bit scale [0,255] for neural network ingestion via the following forward transformation (Equation 7):

$$I_{\text{norm}}=\left(\frac{HU_{\text{orig}}-(-1,024)}{3,000-(-1,024)}\right)\times 255$$ (7)

After generating the synthetic 2D slices, they were stacked back into a 3D volume. To ensure optimal downstream segmentation accuracy, we programmatically resampled the voxel size and realigned the spatial coordinates (affine matrix) to perfectly match the original 3D CT volume. An exact mathematical inverse transformation was then applied to restore pixel intensities to the clinical HU scale (Equation 8):

$$HU_{\text{restored}}\;:\;HU_{\text{restored}}=\left(\frac{I_{\text{gen}}}{255}\right)\times (3,000-(\;\;-1,024))+(\;\;-1,024)$$ (8)

where $I_{\text{gen}}$ denotes the direct pixel intensity output from the generative network, represented as a normalized grayscale value within the standard 8-bit [0,255] domain. >Crucially, while spatial alignment was perfectly restored, the initial 8-bit quantization compresses the CT dynamic range, leading to irreversible fine-grained information loss. Because generative networks prioritize macro-structural features, they subtly smooth low-density ranges. When these minor 8-bit shifts are re-expanded into the true HU scale, the deviations are magnified, pushing fat voxels outside the strict [−190,−30]HU diagnostic window. This underpins the observed downstream compositional collapse.

##### 2.3.2.2 Segmentation Pipeline and Tissue Quantification

The harmonized volumes were processed using TotalSegmentator (16), a robust deep-learning framework that automatically delineates 117 distinct anatomical structures, including major organs, skeletal components, and vascular segments. Simultaneously, we employed a Body Composition Analysis (BCA) module (27) to quantify specific tissue compartments. This module uses standardized Hounsfield Unit (HU) thresholds to distinguish among skeletal muscle, visceral adipose tissue (VAT), subcutaneous adipose tissue (SAT), and intermuscular adipose tissue (IMAT). For each structure, the total volume (mL) and mean HU density were extracted, providing quantitative volumetric measurements for downstream harmonization evaluation.”

##### 2.3.2.3 Evaluation Metrics and Significance

The primary metric for assessing the clinical utility of the harmonized images was the Relative Volume Error (RVE), defined as the percentage deviation of the harmonized volumes from the original reference volumes (reconstructed via IMR and iDose${}^{4}$). The RVE (Equation 9) is calculated as:

$$RVE=\frac{V_{synth}-V_{ref}}{V_{ref}}\times 100\%$$ (9)

where $V_{synth}$ and $V_{ref}$ represent the volumes of a specific structure in the generated and reference CT scans, respectively.

The use of volumetric segmentation as an evaluation metric is critical for three reasons. First, it transcends traditional pixel-level metrics such as PSNR and SSIM, which often fail to capture the clinical validity of medical images. Second, it serves as a high-sensitivity “stress test” for the generative model’s intensity mapping. Third, by comparing these metrics across reconstruction baselines (IMR vs. iDose${}^{4}$), we evaluated the robustness of the synthesis algorithm to variations in the underlying CT reconstruction technology. This comprehensive approach ensures that harmonized images maintain the diagnostic integrity necessary for clinical applications such as opportunistic screening (28) for sarcopenia and metabolic disorders.

## 3 Results

**Figure 6:**
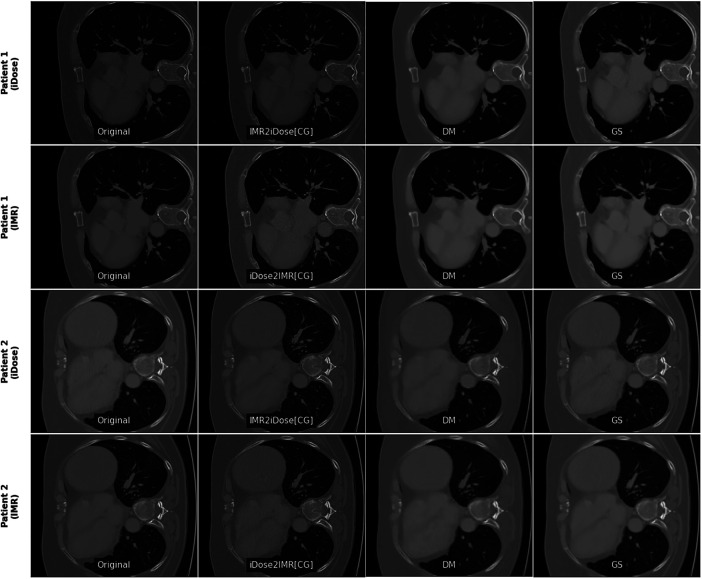
Comparison of image translation and harmonization results for two representative patient slices. To facilitate close visual inspection of fine structural textures while avoiding manual alignment bias, all subplots are programmatically and uniformly cropped using an automated relative bounding box (retaining [20%, 80%] along the vertical Y-axis and [25%, 85%] along the horizontal X-axis of the original field of view). This protocol ensures strict, identical spatial and pixel-level alignment across all methods, reflecting the true underlying affine and orientation correspondence.

We present two sets of paired slices from two different patients, together with the results of I2I translations in Figure 6. Although the figures are not representative, several observations can be made. Due to the grayscale presentation, the main perceived differences are likely to be attributed to sharpness and edge preservation. Hence, the comparison of the data examples to the presented metrics may not be straightforward. IMR reconstruction seems to be more blurry than iDose${}^{4}$, Gaussian Filter, as expected, increases blurriness, while CycleGAN, somewhat unexpectedly, seems to provide a certain level of quality improvement. However, the focus of this work is not on quality assessment but on understanding differences in data distribution.

### 3.1 Performance metrics for “bias reduction potential”

**Figure 7:**
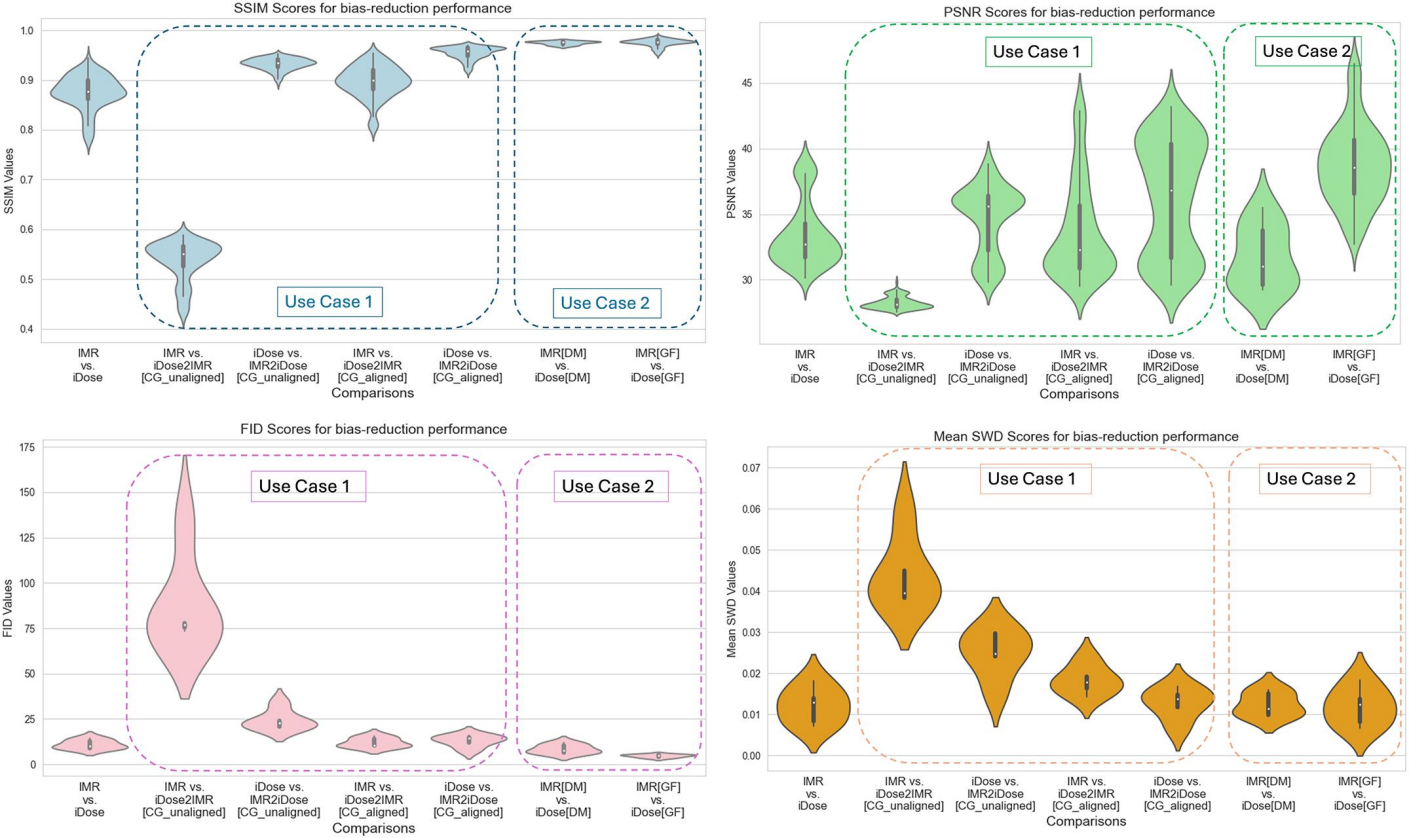
Metric results for “bias reduction potential”.

Distribution comparisons for original data, Use Case 1 (one data set is transformed with a generative approach), and Use Case 2 (both data sets are transformed with denoising methods). In Figure 7, the SSIM and PSNR results illustrate two pixel-to-pixel metrics, while FID and SWD illustrate metrics in feature spaces.

The metrics presented in the figures reflect distributional similarities relevant to the respective Use Cases. The first comparison, between IMR and iDose${}^{4}$, using the original datasets, serves as a baseline. The SSIM distribution in the original comparison between the two reconstruction methods is broad, ranging from approximately 0.75 to 0.95, peaking near 0.87. This indicated a relatively high original similarity but with notable variability in the dataset.

The metric results correspond to Use Case 1, in which transforming one dataset is expected to bring the distributions closer together. By comparing unaligned and aligned training, we observe that the aligned CycleGAN achieves better performance across all metrics. Neither of the two CycleGAN translation directions yields an essential improvement in distributional differences. In Figure 7, the last metric SWD shows a decrease in similarity, although the scale of the differences remains very small.

The second Use Case considers the case in which both data sets are transformed to bring them closer together. Both the Denoising Diffusion Model and the Gaussian Filter are performing well, except for the PSNR score for DM in Figure 7.

### 3.2 Assessment of the transformation range due to the I2I methods

**Figure 8:**
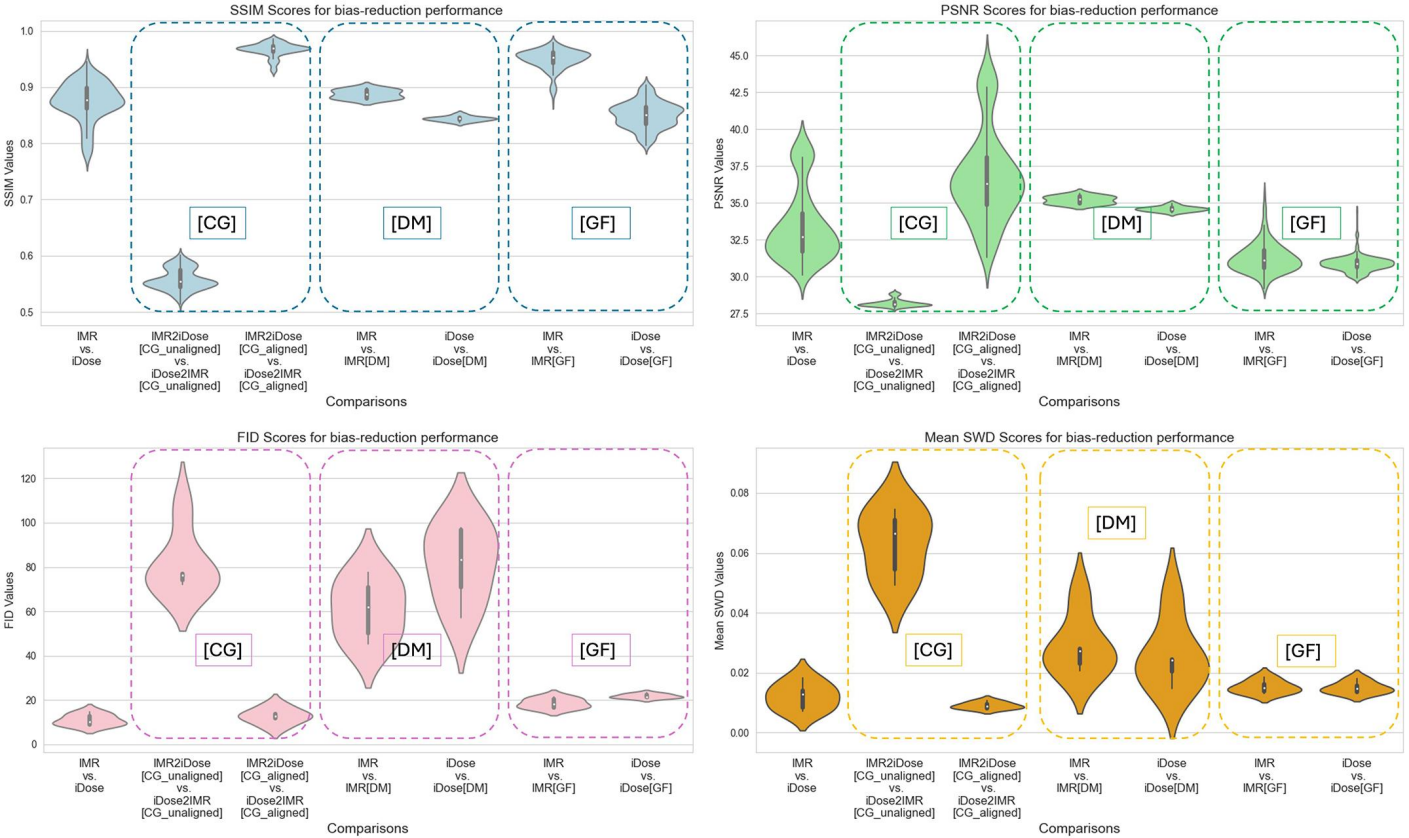
Metric results comparisons for I2I methods.

Distribution comparisons for original data, and the distributions of the data transformed by three different I2I methods: CycleGAN, Denoising Diffusion, and Gaussian Filtering. In Figure 8, SSIM and PSNR illustrate two pixel-to-pixel metrics, and FID and SWD illustrate metrics in feature spaces.

The metrics presented in the Figure 8 provide a deeper understanding of the I2I effects. For example, we observe that the CycleGAN, can bring the distributions closer to each other if applied to both data sets simultaneously. Denoising diffusion leads to quite strong alterations in comparison to the original data sets, particularly in the feature spaces (FID and SWD), and not that strong visible changes (SSIM). On the other hand, from Figure 7, we learn that the alterations to both original datasets bring the distributions closer together across all measured spaces. The Gaussian Filter, although yielding visible changes (as measured by SSIM), does not seem to strongly alter the feature spaces.

### 3.3 Assessment of segmentation results

**Figure 9:**
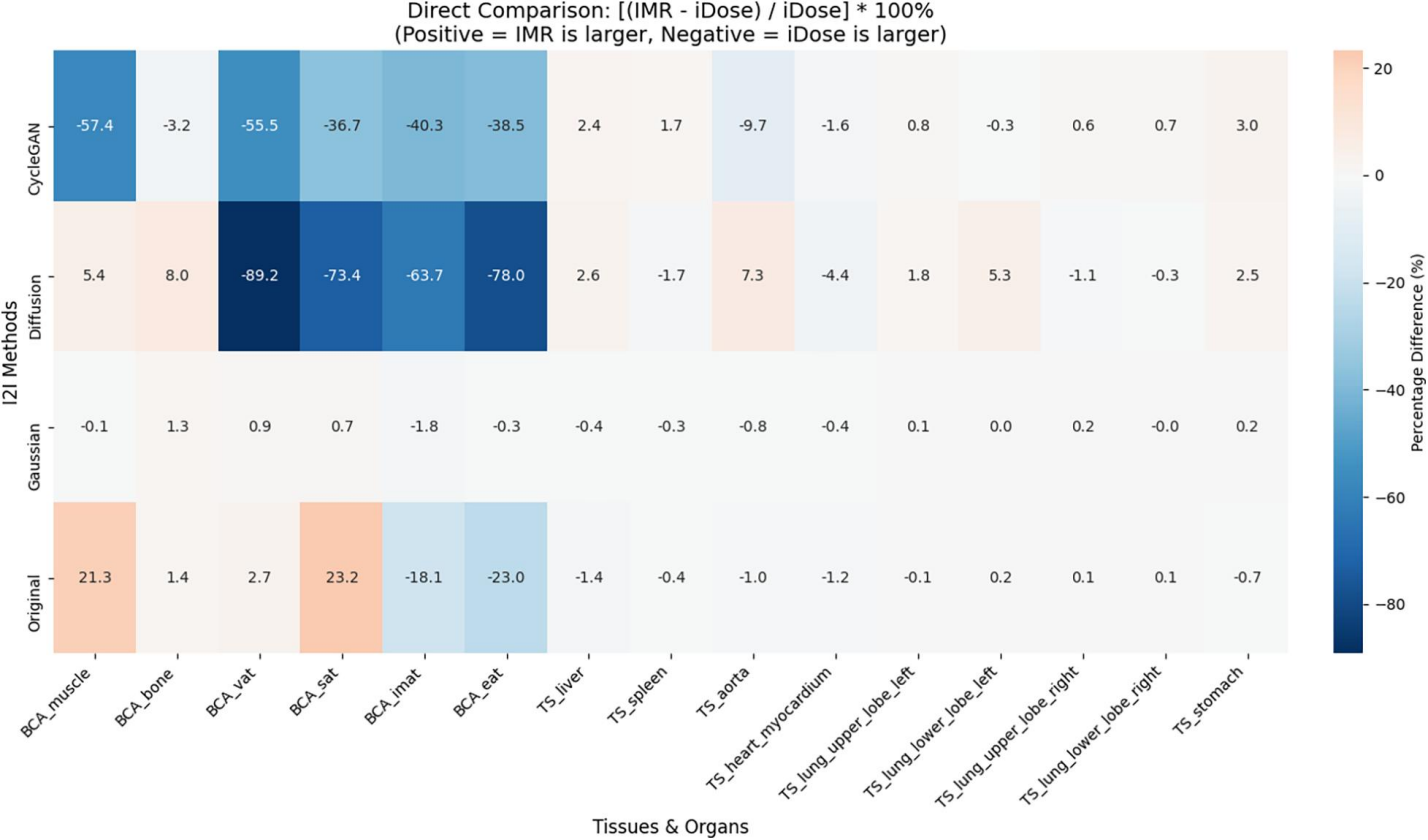
Heatmap detailing the Percentage Volume Difference [(IMR - iDose${}^{4}$) / iDose${}^{4}$] * 100% between harmonized images, showcasing the impact of harmonization on inter-mode systematic bias. The ’Original’ row serves as a baseline comparison of the inherent difference between original IMR and iDose${}^{4}$ volumes. The ’Gaussian’ row serves as a control, exhibiting minimal bias shift (close to 0%), suggesting that it preserves the original anatomically coherent relationships. Conversely, the Diffusion and CycleGAN rows introduce substantial new inter-mode biases, particularly Diffusion’s substantial underestimation of harmonized IMR volumes relative to iDose${}^{4}$ in adipose-related BCA tissues (e.g., −89.2% in VAT), directly illustrating its failure to generalize to fat structures.

In Figure 9, the Gaussian submode demonstrates the highest stability and accuracy, maintaining the lowest Relative Volume Error (RVE) against the original validation data and showing minimal discrepancy (near 0%) between IMR and iDose${}^{4}$ across most tissues.

In contrast, Diffusion and CycleGAN both exhibit severe performance degradation in specific adipose tissues (BCA categories), with RVEs frequently reaching −100%, indicating a failure to preserve volume during the Image-to-Image (I2I) translation.

The Diffusion submode introduces the most significant bias between the two main modes, consistently producing much lower volumes in IMR compared to iDose${}^{4}$ (notably a −89.2% difference in BCA_vat), whereas CycleGAN shows a similar but slightly less extreme trend of underestimation.

**Figure 10:**
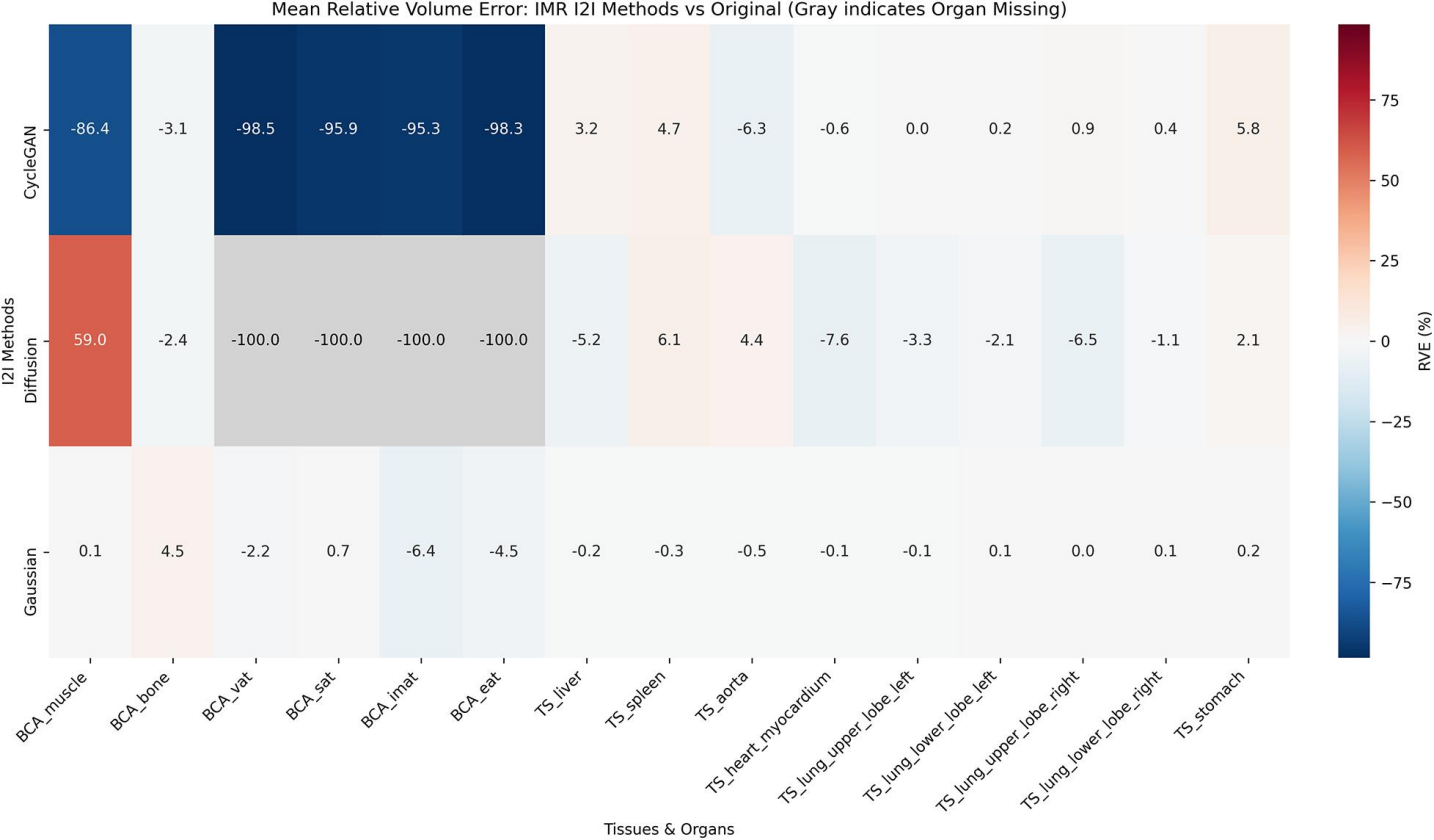
Heatmap illustrating the Mean Relative Volume Error (RVE, %) for multi-organ segmentation based on images harmonized to the IMR (Iterative Model Reconstruction) mode. This plot provides a detailed anatomical analysis of volumetric bias introduced by each Image-to-Image (I2I) translation method (Diffusion, CycleGAN, Gaussian filter) compared to the original IMR validation standard. The Diffusion and CycleGAN models exhibit a catastrophic underestimation of volumes in multiple BCA adipose categories (e.g., −100% in BCA_eat). The near-zero RVE of the Gaussian filter suggests it as a stable and computationally efficient baseline for this harmonization task, warranting further validation.

**Figure 11:**
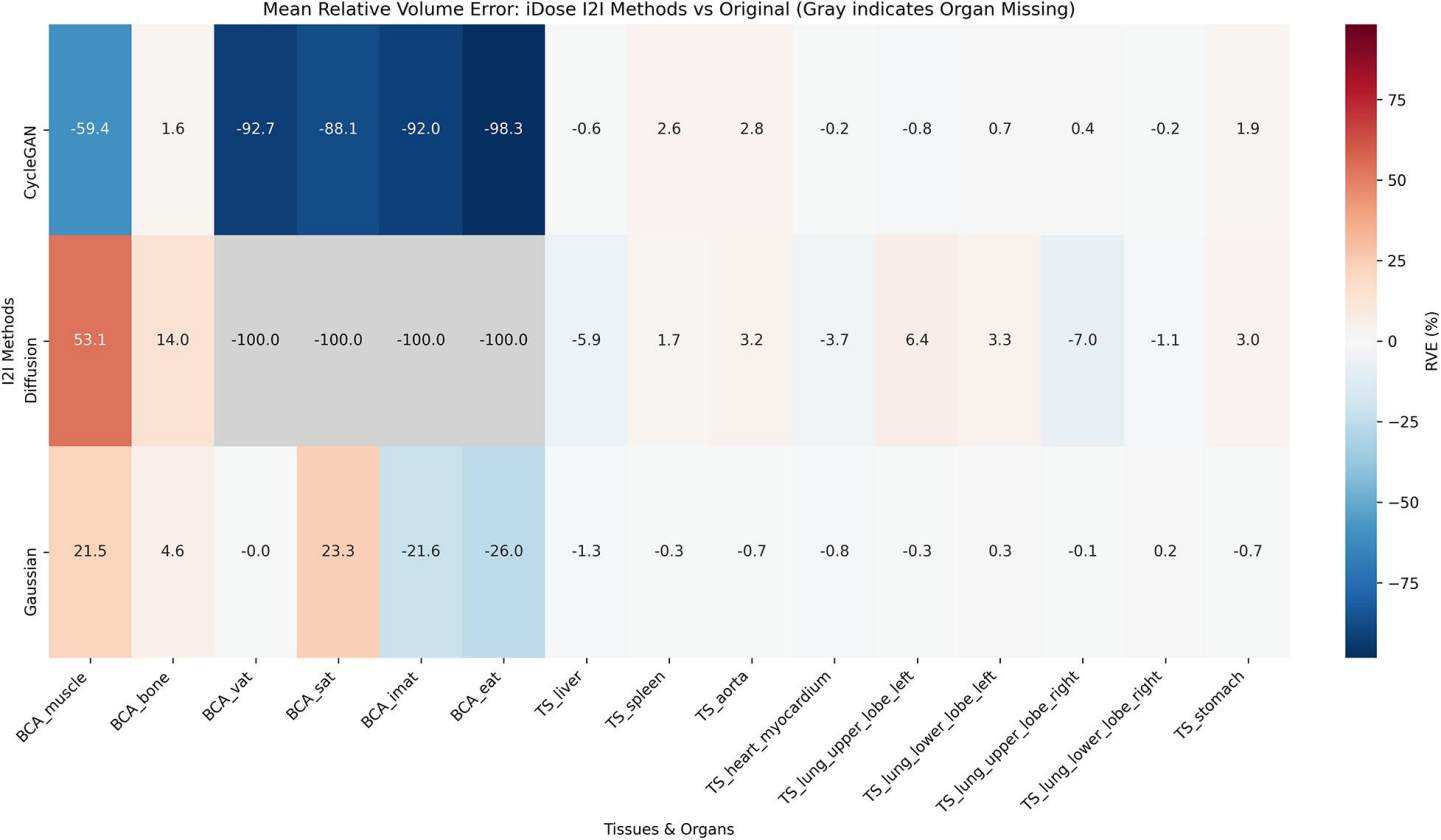
Heatmap of Mean Relative Volume Error (RVE, %) for multi-organ segmentation derived from CT images harmonized to the iDose${}^{4}$ reconstruction mode. The plot quantifies the volumetric deviation of generated images (by Diffusion, CycleGAN, and Gaussian filter) from the original iDose${}^{4}$ validation data across multiple anatomical structures, including Body Composition Analysis (BCA) tissues and general organs. Crucially, extreme negative values, reaching −100% in adipose-related BCA categories for the Diffusion model (VAT, SAT, IMAT, EAT), highlight a complete failure in structural preservation.

However, all three methods yield acceptable results for organ segmentation. For the comparison of each mode, as shown in Figures 10 and 11, similar patterns are illustrated on the RVE results.

The quantitative analysis of the harmonized CT images revealed a failure to preserve visceral adipose tissue (VAT) across the deep generative methods (Diffusion and CycleGAN). While large anatomical structures such as the liver and spleen maintained relatively stable volumes, the Relative Volume Error (RVE) for VAT reached −100% across the majority of generative samples.

## 4 Discussion

Our study performed a pairwise evaluation of Image-to-Image (I2I) translation methods to reduce distributional distances between CT images reconstructed via IMR and iDose${}^{4}$. As noted (12), inter-vendor and inter-reconstruction differences necessitate robust harmonization before deploying clinical AI. Using a paired dataset enabled us to directly measure distribution shifts, revealing that while task-agnostic metrics (FID, SWD) serve as useful indicators of feature-level bias, they do not consistently predict downstream performance.

The summary Table 1 illustrates following points: The Gaussian Filter demonstrated strong stability, offering simplicity, interoperability, and high similarity in feature distributions. However, its tendency to blur structural edges necessitates careful validation, as segmentation tasks are inherently more sensitive to boundary sharpness than density-based assessments.

**Table 1:** Summarized comparison of methods.

Property	CycleGAN	Denoising Diffusion	Gaussian Filter
Improves distributions in pix2pix	0	+	++
Improves distributions in features	++	0	+
Computation speed	+	−	++
Segmentation performance	−	−	+

− minimal; 0 unclear; + average; ++ fast/strong.

In contrast, while Denoising Diffusion achieved strong feature-level distribution alignment in specific configurations, its high computational cost requires further parameter optimization to justify its use in efficient AI frameworks for radiology.

CycleGAN appeared to perform worse on pixel-to-pixel metrics, likely because it prioritizes independent feature enhancement over strict distributional alignment. The asymmetry between IMR2iDose${}^{4}$ and iDose${}^{4}$2IMR translation directions likely reflects the directional nature of the learned mapping, which may not be equally well-constrained in both directions, given the relatively small training set. Nevertheless, our use of paired data provided a significant advantage over typical unpaired CycleGAN implementations by ensuring anatomical consistency and minimizing confounding inter-patient variability, both of which are critical for fine-grained anatomical fidelity.

The low PSNR score observed for the Diffusion Model in Use Case 2 may reflect a trade-off between distribution alignment and pixel-level fidelity; however, feature-level metrics indicate that the images’ informative content remains comparable, suggesting this does not constitute a clinically meaningful loss of quality.

The most striking finding was the failure in Body Composition Analysis (BCA), specifically the −100% RVE in visceral adipose tissue (VAT). Despite high visual fidelity, generative models induced a “systematic anatomical erasure.” This is primarily attributed to the Hounsfield Unit (HU) shifting. When images are normalized to a standard [0, 255] range for generative training, the precise densitometric integrity of CT is compromised. Even minor intensity shifts can push low-density fat pixels outside the strict diagnostic window (−190 to −30 HU), causing downstream tools like TotalSegmentator to misclassify these regions. This mechanism explains the −100% RVE observed in the Results: the BCA pipeline failed to identify any visceral fat voxels within the predefined HU window, while the original IMR and iDose${}^{4}$ reference volumes remained consistent. Furthermore, the 3D inconsistency arising from stacking 2D-generated slices degrades high-frequency features and boundary sharpness, which are necessary for distinguishing thin adipose layers from adjacent tissues. In addition, the previous 3D patch-based generative models (19) perform undesirably. The underperformance of generative approaches is therefore likely attributable to suboptimal model adaptation—specifically, the intensity normalization mismatch and the absence of 3D-aware architectures—rather than to inherent limitations of I2I methods, which retain significant potential to mitigate reconstruction-related bias. Hence, for a 3D generative model, a high-resolution scaling model might generalize better to organs and tissues.

A primary limitation of this study is the small cohort size, comprising only five pairs of patients. Although using 1,491 2D slices provides a substantial sample size for image-texture and pixel-level analysis, it does not overcome the inherent lack of clinical and anatomical diversity at the patient’s level. Consequently, the generalizability of our findings to a broader, more heterogeneous clinical population remains to be fully validated. However, as a proof-of-concept study focused on technical harmonization, this paired design successfully demonstrates the feasibility and potential pitfalls of 2D I2I pipelines. Future research will focus on scaling this validated technical pipeline to larger, multi-center datasets to comprehensively test its robustness across diverse patient demographics and clinical pathologies. To address the dual challenges of multicenter data heterogeneity and patient privacy, moving beyond centralized generative models towards decentralized paradigms represents a critical next step. As suggested by Li et al. (29), a promising approach is to leverage distributed network infrastructure—such as Federated Learning—to enable robust data translation and domain harmonization while strictly preserving data sensitivity. Integrating such privacy-preserving distributed frameworks with resource-efficient translation pipelines could eliminate center-specific reconstruction biases without requiring the risk-prone pooling of raw clinical data.

Furthermore, our findings reveal a profound methodological decoupling between task-agnostic image quality metrics (such as SSIM and PSNR) and clinical performance metrics (such as RVE). Statistical correlation analysis between these two tiers demonstrates no meaningful linear relationship. This absence of correlation stems from the non-linear and localized failure modes characteristic of deep generative models. A synthetic image can achieve near-perfect macro-structural fidelity and visual plausibility, yields high SSIM and low FID scores by accurately preserving dominant anatomical structures. Yet, a minute, uniform Hounsfield Unit (HU) shift within a low-contrast sub-domain can completely push specific tissues—such as visceral adipose tissue—outside their diagnostic segmentation windows. This results in an abrupt, localized −100% RVE (’compositional collapse’) that remains mathematically invisible to global image-similarity metrics. This critical discrepancy firmly underscores the core thesis of this study: standard, task-agnostic image evaluation benchmarks are fundamentally insufficient and potentially deceptive when validating AI-driven image harmonization for quantitative radiology.

## 5 Conclusion

The reported results suggest that in the context of efficient AI for radiology imaging—where efficiency encompasses not only computational cost but also reliability of clinical outputs—standard image-quality metrics are insufficient proxies for downstream clinical performance. High-parameter generative models may introduce structural losses that remain invisible to such metrics yet critically affect clinical utility. The underperformance of generative approaches observed here is likely due to suboptimal model adaptation rather than an inherent limitation of I2I methods, which retain significant potential for mitigating reconstruction-related bias in radiology AI. Future work developing harmonization pipelines must therefore prioritize task-specific, clinically grounded validation as the primary benchmark—and carefully consider whether computationally simpler approaches, such as conventional filtering, may already provide comparably stable harmonization for specific use cases before resorting to high-parameter generative solutions.

Future work will address the key limitations identified in this proof-of-concept study. First, dataset expansion through larger patient cohorts is necessary to provide sufficient training data for high-parameter generative models, whose performance is particularly sensitive to data volume. Second, parameter optimization—specifically refining intensity normalization and training procedures to preserve high-contrast diagnostic information in the HU range critical for body composition analysis—is required before generative I2I methods can be considered reliable for clinical harmonization. Third, and most importantly, task-specific downstream validation must be established as the primary benchmark for evaluating harmonization quality, replacing or complementing task-agnostic image-quality metrics. Together, these steps will determine whether generative I2I approaches can meet the dual efficiency criterion—computational feasibility and clinical reliability—that this work has identified as essential for deployment in radiology AI pipelines.

## Data Availability

The original contributions presented in the study are included in the article/Supplementary Material, further inquiries can be directed to the corresponding author/s.
